# Enterococcal Cerebellopontine Angle Abscess in a 12-year-old Female

**DOI:** 10.4103/0974-777X.59255

**Published:** 2010

**Authors:** Alka Sonavane, Vasant Baradkar, Simit Kumar

**Affiliations:** *Department of Microbiology, Lokmanya Tilak Municipal Medical College & General Hospital, Sion, Mumbai - 400 002, India*

**Keywords:** Brain abscess, Computerized tomography, *Enterococcus species*

## Abstract

Despite advances in imaging and antibiotic treatment, brain abscess is still encountered occasionally. Various aerobic and anaerobic bacteria have been reported as causative agents of brain abscess but only a few cases of enterococcal brain abscesses have been reported. Here we report a case of brain abscess in a 12-year-old female patient, who presented with a history of fever, chills, headache, convulsions since seven days and history of altered sensorium and aphasia since the last two days. The patient had chronic suppurative otitis media of both ears following trauma and presented with ear discharge. The diagnosis of brain abscess was done by computerized tomography scan and the pus was aspirated by left suboccipital burr hole operation. *Enterococcus species* was cultured from the aspirated pus sample. The patient responded to surgical drainage and antibiotic treatment.

## INTRODUCTION

Despite the advent of modern neurosurgical techniques, new antibiotics and new powerful imaging technologies, brain abscess remains a potentially fatal central nervous system (CNS) infection.[1–4] Associations for portal of entry include hematogenous spread, postneurological states, contiguous infection from parameningeal foci such as otogenic origin and paranasal sinusitis.[1] The causative pathogens of bacterial brain abscess vary with time period, geographic distribution, age, underlying medical and/or surgical conditions and mode of infection. A large number of Gram positive cocci, Gram negative aerobic bacilli, anaerobes and *Mycobacterium tuberculosis* have been reported as the causative agents of bacterial brain abscess.[1–7] Only a few cases of brain abscess due to *E. species* have been reported.[2–4] We report a rare case of enterococcal brain abscess in a 12-year-old female child, which was managed successfully with surgical drainage and antibiotics.

## CASE REPORT

A 12-year-old female patient presented with history of fever with chills and headache, convulsions since last seven days and a history of altered sensorium and aphasia since the last two days. The child had a past history of trauma to the right ear at the age of four years, which was followed by bleeding, which at that time stopped spontaneously, but there was a history of intermittent discharge from the right ear since the last three years and she had a similar discharge from the left ear since the last six months. There was no history of nystagmus, vertigo or any other major illness in the past. On examination the patient was febrile, pulse of 100/minute, blood pressure of 100/80. The patient was drowsy, both the pupils were dilated, reacting sluggishly to light. The plantar reflex was increased on both sides. The findings of cardiovascular and respiratory system were within normal limits. Perabdominal examination was also within normal limits. There was discharge from both the ears.

**Figure 1:**
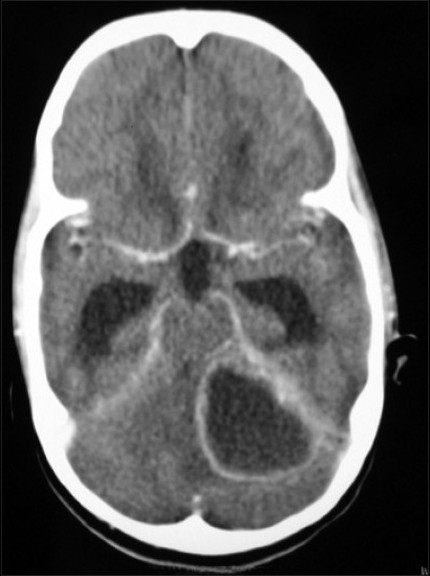
Hypodense extra axial lesion with peripheral rim enhancement measuring approximately 4.4 × 3.5 × 3.0 cm suggestive of abscess

Her investigations revealed that the hemoglobin was 11.8 g/dl, the total leukocyte count was 6900/mm^3^ with polymorphs 89%, platelets 3,50000/mm^3^, fasting blood sugar was 81 mg/dl and postprandial levels were 110 mg/dl, Na^+^ - 136 mEq/L, K^+^ - 2.4 mEq/L. The total bilirubin was 0.6 mg%. Computerized tomography (CT) showed hypodense extra axial lesion with peripheral rim enhancement measuring approximately 4.4 × 3.5 × 3.0 cm suggestive of a brain abscess [Figure 1]. Hypodense soft tissue was seen in the left mastoid air cells and middle ear cleft with erosion of the underlying tegmen and sinus, however no midline shift was noted. There was an abnormal hypodense non-enhancing filling defect seen in the left sigmoid sinus, suggestive of a thrombus and features suggestive of active obstructive hydrocephalus. The blood cultures collected from different sites on different occasions showed no growth in culture.

**Figure 2:**
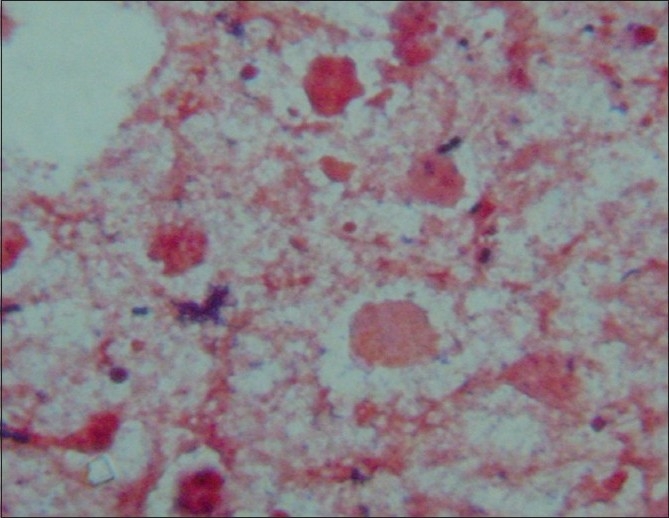
Gram stained smear showing oval, Gram positive cocci in pairs - arranged end to end

The cerebellopontine abscess was drained under anesthesia by retromastoid burr hole operation. The drained abscess material was processed for Gram staining, Ziehl Neelsen staining and was cultured aerobically on blood agar, chocolate agar, MacConkey agar, Lowenstein Jensen medium, Sabouraud's dextrose agar and thioglycollate broth. The Gram stained smear showed pus cells with Gram positive oval cocci in pairs with typical arrangement suggestive of *E. species* [Figure 2]. After overnight aerobic incubation α hemolytic colonies were observed. The isolate was identified as *E. species* and was sensitive to vancomycin, ciprofloxacin and amoxycillin + clavulanic acid. Meanwhile the patient was started on Vancomycin and Ceftriaxone. The patient responded to the surgical drainage and the antibiotics, with the rapid improvement in the neurological status. The patient was later discharged after receiving three weeks of intravenous antibiotics and was discharged on oral Ciprofloxacin for one week. The patient on follow up visits was doing well with no residual neurological deficit and marked improvement in the radiological findings on a follow-up CT scan.

## DISCUSSION

There have been many advances with respect to the diagnosis and management of brain abscess, resulting in a corresponding increase of survival rates. However its incidence is high, approximately 5% per million people, and the number of immuno-deficient hosts having high risk of opportunistic infections might be increasing. This disease continues to be one of the most common neurosurgical diseases.[1–5]

Criteria[1] for inclusion of bacterial brain abscess are:

- Characteristic computerized tomography and/or magnetic resonance imaging findings
- Evidence of brain abscess during surgery or histopathological examination
- Classical manifestations including fever, headache, localized neurological signs and/or consciousness disturbances.

All these criteria are met in the present case.

Regarding portal of entry, brain abscess is almost always secondary to a focus of suppuration elsewhere in the body or may develop either by a contiguous focus of infection, head trauma or hematogenous spread from a distant focus.[1–5] Thus the predisposing factors for the development of brain abscess include infections of the middle ear, mastoid, paranasal sinuses, orbit, face, scalp penetrating skull injury, intracranial surgery including insertion of ventriculo-peritoneal shunts.[5–6] Otitis media is common in India.[2–6] A previous study conducted by Malik *et al*.[6] from Mumbai, showed that in 47 cases of brain abscess, the primary focus of infection could be established in 37 cases (78.7%) and otogenic source was the commonest in 34% cases. Another prospective study of pattern of brain abscess reported from India[7] showed chronic suppurative otitis media to be the commonest predisposing factor in 48% of patients. In this case too, the patient presented with chronic suppurative otitis media following trauma, which acted as a source of infection for the brain abscess.

The location and number of abscesses depends upon the predisposing factors. The temporal abscess and the cerebellum are the commonest sites following otogenic source as observed in the present case. The list of bacteria causing brain abscesses is very large. It includes Gram negative aerobic bacilli like *Klebsiella pneumoniae, Pseudomonas aeruginosa, Esherichia coli, Salmonella* species, *Proteus* species*, Klebsiella oxytoca, Haemophilus influenzae, Pasturella* species*, Vibrio cholerae* non 01; Gram positive bacteria like *Staphylococcus aureus,* other *Staphylococcus* species*, Streptococcus pneumoniae, E. species, Viridans streptococci,* other *Streptococcal* species. The anaerobic organisms reported are *Bacteroides* species*, Fusobacterium* species*, Peptostreptococcus* and *Propionibacterium* species*.*[1–4]

The commonest organisms causing brain abscess following an otogenic source include *Staphylococcus aureus, Streptococcus pneumoniae, H. influenzae, E.coli, Ptoteus* and *Pseudomonas* species.[1–7] Very few studies have reported *E. species* as one of the causative agents of brain abscesses in India. Kurien *et al*.[2] in 1993 reported *E. species* as one of the causative agents of brain abscesses in India, while studying 153 cases of brain abscesses. In the year 2002, Park *et al*.[4] from Korea reported a case of otogenic brain abscess due to *E. species*. A recent case report from India by Mohanty *et al*.[3] in the year 2005 reported a brain abscess due to *Enterococcus avium* in a 19-year-old man with chronic otitis media since childhood. The patient presented to the emergency department in a comatose condition. Contrast enhanced computerized tomography of the brain showed a brain abscess in the right temporal lobe. Emergency temporal burr hole operation was performed and the pus drained out yielded *Enterococcus avium* in culture.

Computerized tomography is a very sensitive and noninvasive tool.[1–6] Indeed CT scan has contributed significantly in reducing mortality both by way of early detection as well as by permitting precise CT guided aspiration in selected cases. In order to differentiate brain abscess from other closely resembling lesions, like tumors, infarcts, hematoma and other space occupying lesions, CT scan acts as an important diagnostic tool.

Surgical drainage and/or incision along with aggressive antibiotic therapy remains the definitive treatment for brain abscess. Some studies utilizing aspiration as only surgical intervention have excellent results and this has been advocated as the first line therapy. Conservative therapy alone is recommended in selected cases, as in early stages of cerebritis, abscess less than 2 cm, surgically inaccessible or multiple abscesses and in patients who are at high surgical risk.[1–6] But surgical treatment along with antibiotics is the preferred regimen nowadays, which helps in reducing mortality.

We would like to stress that a high index of suspicion, timely diagnostic support by CT scan, surgical intervention and vigorous antimicrobial therapy are crucial for better outcome.
